# Supplementary material for: A 14-Day Plant-Based Dietary Intervention Modulates the Plasma Levels of Rheumatoid Arthritis-Associated MicroRNAs: A Bioinformatics-Guided Pilot Study
Source: Nutrients. 2025 Jul 4;17(13):2222. doi: 10.3390/nu17132222 (PMC12251866; doi:10.3390/nu17132222)
Supplement: Supplementary file 1 [file nutrients-17-02222-s001.zip › nutrients-3714764-supplementary.pdf]

Supplementary Table S1. Lifestyle variables (smoking and alcohol consumption), RA drug treatments, and percent change in clinical parameters at the patient level

			**csDMARDs**						**tsDMARDs**	**NSAIDs**			**Steroids**			
Patient	Smoking	Alcohol consumption	Methotrexate	Leflunomide	Sulfasalazine	Chloroquine	Hydroxychloroquine	Azathioprine	Baricitinib	Etoricoxib	Ibuprofen	Celecoxib	Prednisone	%Δ DAS28-CRP	%Δ CRP	%Δ ESR
P01	No	Yes	Yes	No	Yes	No	No	No	No	No	No	No	Yes	76.3	53.79	100
P02	No	Yes	Yes	Yes	Yes	No	No	Yes	No	No	No	No	No	75.37	25	215.79
P03	Yes	No	Yes	Yes	Yes	No	No	No	No	No	No	No	No	72.14	47.8	107.14
P04	No	No	No	No	Yes	No	No	No	No	No	No	No	No	79.38	97.79	125
P05	No	No	Yes	No	Yes	No	No	No	No	No	No	No	Yes	71.43	87.02	80
P06	No	No	Yes	No	No	No	Yes	No	No	Yes	No	No	No	79	90	150
P07	No	No	Yes	No	No	No	Yes	No	No	No	No	No	No	100	13.78	65
P08	No	No	Yes	No	No	Yes	No	No	No	No	No	Yes	No	79.86	92.01	75
P09	Yes	No	Yes	No	No	No	No	No	No	No	No	No	No	81.63	87.51	105.88
P10	No	No	Yes	No	Yes	No	Yes	No	No	No	No	No	No	74.3	85.2	96.55
P11	No	No	Yes	No	Yes	No	No	No	No	No	No	No	No	84.9	85.2	122.22
P12	No	No	Yes	No	No	No	No	No	No	No	No	No	Yes	83.08	30.34	118.03
P13	No	No	No	No	Yes	No	No	No	No	No	No	No	No	84.74	58.01	77.78
P14	Yes	No	Yes	No	No	No	No	No	No	No	No	No	Yes	104.62	78.03	63.16
P15	No	No	Yes	No	Yes	No	No	No	No	No	No	No	Yes	99.45	87.95	77.78
P16	No	No	Yes	No	No	No	No	No	No	Yes	No	No	No	96.89	96.76	87.5
P17	No	Yes	Yes	No	No	No	No	No	Yes	No	No	No	Yes	103.04	174.62	163.64

P18	No	No	Yes	Yes	Yes	No	No	No	No	No	No	No	No	80.26	23.46	57.89
P19	No	No	Yes	No	No	No	Yes	No	No	No	No	No	No	41.01	10.2	100
P20	No	No	Yes	No	Yes	No	Yes	No	No	No	No	No	No	80.35	74.18	114.29
P21	No	No	Yes	Yes	No	No	No	No	No	No	No	No	No	93.11	10.2	166.67
P22	No	No	No	Yes	Yes	No	No	No	No	No	No	No	No	-	11.14	112.5
P23	No	No	Yes	Yes	No	No	Yes	No	No	No	No	No	No	-	-	-

csDMARDs: conventional synthetic DMARDs

tsDMARDs : targeted synthetic DMARDs

%Δ DAS28-CRP: Percent change in Disease Activity Score 28 based on C-reactive protein

%Δ CRP: Percent change in C-reactive protein

%Δ ESR: Percent change in erythrocyte sedimentation rate

Supplementary table S2. miRNAs involved in signaling pathways related to Rheumatoid Arthritis

miRNA ID	P-value post PBD	Biological significance	Pathway Involved	References
hsa-miR-16-5p	0.4100	Involved in cellular processes related to inflammation	regulators of chondrocyte maturation and proliferation	[35]
hsa-miR-26a-5p	0.0192	Enhances cells proliferation, invasion, and apoptosis resistance of fibroblast-like synoviocytes	PTEN/PI3K/AKT pathway	[33]
hsa-miR-125a-5p	0.0374	Associated with markers of inflammation	JAK-STAT signaling pathway	[37]
hsa-miR-125b-5p	0.1001	Regulates cell growth, proliferation, survival and inflammation.	PI3K/Akt/mTOR signaling pathway	[38]
hsa-miR-146a-5p	0.1297	Tumor necrosis factor-alpha	NF-κB signaling pathway	[39]
hsa-miR-155-5p	0.0284	Involved in cellular processes related to inflammation	IRF2BP2/KLF2/NF-κB pathway	[40]
